# High-need dental deserts in England: a national spatial analysis of NHS access and deprivation

**DOI:** 10.1038/s41415-026-9877-2

**Published:** 2026-08-14

**Authors:** Hugh Devlin

**Affiliations:** University of Bristol, Bristol, United Kingdom

## Abstract

**Aims** The aim of this study was to identify high-need dental deserts in England and quantify the population affected and profile the demographics relative to other deserts.

**Methods** Lower Layer Super Output Area **(**LSOA) December 2021 boundaries were linked to Index of Multiple Deprivation (IMD) 2025 (deciles; mid-2022 population). National Health Service (NHS) dental practices were geocoded using the ONS Postcode Directory (British National Grid). Euclidean distance from each LSOA centroid to the nearest NHS-listed practice was calculated; LSOAs with no provider within 5 km were classified as dental deserts. High-need deserts were deserts in IMD deciles 1–3. Demographic profiling used Census 2021 ethnicity (TS021) and age structure (TS007a).

**Results** Across England, 1,340 LSOAs (population 2,342,985) had no NHS-listed dental provider within 5 km. The high-need deserts comprised 112 LSOAs (189,542 residents). A further 2,153,443 residents in the IMD 4–10 group lived in dental deserts. The high-need desert population was predominantly white (97.9%) and had a similar age structure to other deserts not in the IMD 1–3 group (0–15: 19.4% versus 19.5%; 65+: 27.4% versus 26.9%).

**Conclusion** Geographic exclusion from NHS-listed dental provision affects ~2.3 million residents. Spatial audits linking deprivation and access could support targeted service planning.

## Key points

- Around 2.34 million people in England live in areas with no NHS dental provider within 5 km.
- The most deprived population (in IMD 1–3) affected by poor access is concentrated not in inner cities, but in rural areas.
- Commissioners should combine small-area deprivation and distance data to identify rural communities where targeted outreach, satellite clinics and workforce incentives are most likely to reduce inequity.

## Introduction

The term ‘dental desert' has been used to describe those areas of the country lacking in a sufficient provision of dental services. A recent (2025) report from the Local Government Association (LGA)^1^ showed considerable variation in the availability of services. Local authorities with higher levels of overall deprivation were more likely to have fewer active National Health Service (NHS) dental practices per 100,000 people than more affluent areas of England and Wales. The LGA report of 2022 found that deprived and rural local authority areas have fewer NHS dentists than those in more affluent urban areas.^2^ It is known that the South West of England, Yorkshire and the Humber and the North West, are most severely affected (98% of practices there do not accept new adult NHS patients).^3^

A dental desert was defined in this study as an area with no NHS dental providers within 5 km of that area's geometric centre. A deprived subgroup of this population (high-need dental desert) was also obtained. The number of people living in England's dental deserts, their geographic distribution and ethnic profile are not known. This study was undertaken to help fill that gap, providing evidence that can inform policy and stimulate debate.

## Methods

### Study design and setting

A cross-sectional spatial analysis was carried out of geographic access to dental providers across England, using Lower Layer Super Output Areas (LSOAs; December 2021 boundaries) as the unit of analysis. LSOAs are small areas designed by the Office of National Statistics (ONS) and contain about 1,000–3,000 individuals. LSOAs are a standard ONS geography designed for statistical reporting; they have consistent coverage across England and are widely used in public health.

LSOAs are small enough to capture within-area inequalities that would be masked at local authority level. The English LSOA boundaries were obtained from the ONS Open Geography Portal (LSOA December 2021, EW, BGC). The socioeconomic deprivation and population data from each LSOA were taken from the Index of Multiple Deprivation (IMD) 2025 File Seven. The IMD divides all English neighbourhoods into ten equal-sized groups based on their level of deprivation, with deciles 1–3 specified as ‘high-need'. The IMD File Seven mid-2022 population denominator was used to estimate the number of residents living in areas meeting dental desert criteria.

Dental providers were derived from an NHS dental practice dataset (‘NHS dental practices from NHS England'). Practices were restricted to those marked ACTIVE where status was available. Accordingly, the provider set represents NHS-listed provision, rather than a complete census of all practices. Provider postcodes were standardised to uppercase and stripped of whitespace and linked to geographic coordinates using the ONS Postcode Directory (ONSPD) hosted table. Coordinates were obtained as British National Grid (OSGB36; EPSG:27700) eastings and northings. Practices without valid coordinates after linkage were excluded from distance calculations.

Geographic access was measured by calculating the planar Euclidean distances from each LSOA centroid to provider locations in the British National Grid coordinate system (EPSG:27700). The Euclidean distance from each LSOA centroid to the nearest dental provider was computed, and the number of providers that were within a 5 km radius of each centroid was determined. This enabled identification of dental deserts, defined as LSOAs with zero dental providers within 5 km of the LSOA centroid. A ‘high-need dental desert' subgroup was defined as LSOAs in IMD deciles 1–3 with no providers within 5 km. The population residing in dental deserts and in the high-need subgroup was estimated. To assess deprivation patterning, outcomes were compared for IMD 1–3 with IMD 4–10, reporting the proportion of each group's population living in LSOAs with no provider within 5 km.

LSOA-level population characteristics were used to profile areas meeting the high-need dental desert definition (IMD 2025 deciles 1–3 and no NHS-listed dental provider within 5 km of the LSOA centroid) and to compare them with other desert and non-desert areas. Ethnicity was obtained from Census 2021 table TS021 and joined to the analytic dataset using LSOA 2021 codes. Ethnic group was collapsed into five broad categories: white, Asian, Black, mixed, other.

Age structure was obtained from Census 2021 table TS007a and aggregated into three bands (0–15, 16–64 and 65+). For age, proportions were calculated using Census 2021 usual resident denominators. Mean age was approximated from Census age-band counts by assigning each age band its midpoint (e.g., 20–24 → 22.5) and calculating a weighted average using the number of people in each band. For the open-ended top band (e.g., 90+), an upper cap of 95 years was assumed so a midpoint could be defined (treating 90+ as 90–95). Comparisons were made between:

- High-need dental deserts (IMD 1–3 and no provider within 5 km)
- Other dental deserts (IMD 4–10 and no provider within 5 km)
- Other deprived areas (IMD 1–3 with at least one provider within 5 km).

Percentages were calculated within each comparison group.

**Fig. 1:**
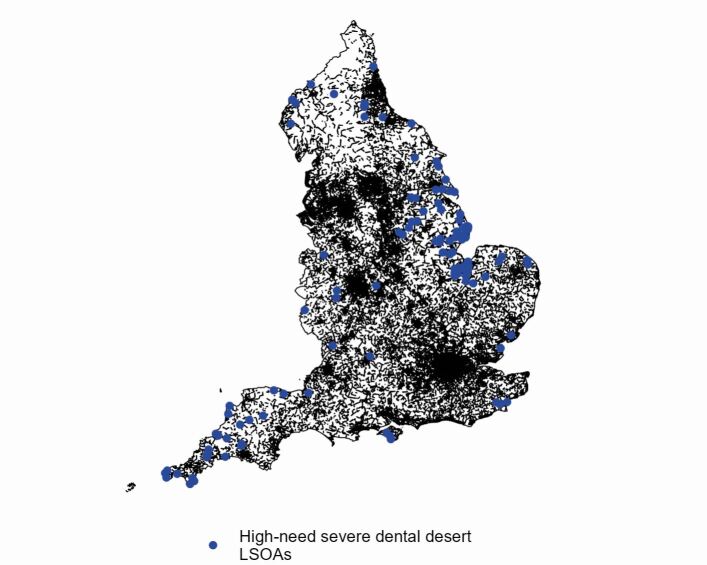
High-need dental deserts in England. These include LSOAs with high need (IMD decile 1–3) and no NHS provider within 5 km represented by blue dots

Figure 1 was constructed by linking LSOA December 2021 boundaries for England to IMD 2025 deciles and an NHS dental practice dataset. Active practices were geocoded from postcodes to British National Grid coordinates using the ONS Postcode Directory. For each LSOA, the centroid was calculated and Euclidean distance to the nearest NHS-listed practice was derived. High-need dental deserts were defined as LSOAs in IMD deciles 1–3 with no NHS-listed practice within 5 km (operationalised as nearest-provider distance >5 km). The centroids of these LSOAs were plotted as blue points over an outline of England.

All data involved in the study was anonymous and publicly available and there was no primary data collection; ethics approval was not required.

### Study geography and linkage completeness

A total of 33,755 English LSOAs were included. IMD 2025 deciles and the mid-2022 population denominator were complete for all included LSOAs. The provider file contained 9,799 active practices after cleaning; 9,791 (99.9%) were successfully geocoded to valid British National Grid coordinates via ONSPD and included in distance calculations.

## Results

Using the criterion of no provider within 5 km, 1,340 LSOAs (4.0% of English LSOAs) were classified as dental deserts, affecting 2,342,985 residents. High-need dental deserts (defined as IMD 1–3 and no provider within 5 km) comprised 112 LSOAs and affected 189,542 residents (Table 1).

**Table 1:** Population living in LSOAs with no dental provider within 5 km, by deprivation group (England)

**IMD group**	**Total LSOAs**	**Total population**	**LSOAs with no provider within 5 km**	**Population with no provider within 5 km**	**% of England population**
IMD 1–3	10,126	17,339,310	112	189,542	0.33%
IMD 4–10	23,629	39,772,819	1,228	2,153,443	3.77%
All IMD deciles (1–10)	33,755	57,112,129	1,340	2,342,985	4.1%
IMD, index of multiple deprivation; LSOA, lower layer super output area.					

High-need dental deserts (IMD deciles 1–3) were less common among the most deprived LSOAs than among the more affluent IMD deciles 4–10. In IMD deciles 1–3, 189,542 inhabitants had no provider within 5 km, representing 1.09% of the IMD 1–3 population. In IMD deciles 4–10, there were 2,153,443 people with no provider within 5 km representing 5.41% of the IMD 4–10 population. Overall, 4.10% of the English population lived in LSOAs with no provider within 5 km.

The high-need dental desert group comprised 189,542 residents which were predominantly white (97.9%), with small proportions identifying as Asian, Black, mixed, or other ethnic groups. This contrasts with other deprived areas (IMD 1–3 LSOAs not in the desert group), which were substantially more ethnically diverse (70.1% white). In IMD deciles 4–10, 85.7% of residents were white. This indicates that the deprived communities experiencing the most geographic isolation from NHS-listed dental provision are demographically distinct from deprived areas overall.

The age structure of the population living in LSOAs with no NHS provider within 5 km (Table 2) was similar in high-need severe deserts (IMD 1–3) and in other severe deserts (IMD 4–10).

**Table 2:** Age structure of high-need dental desert population (IMD 1–3) compared with the more affluent dental desert population (IMD 4–10)

**Desert stratum**	**LSOAs (n)**	**Census 2021 usual residents (n)**	**0–15 years n (%)**	**16–64 years n (%)**	**65+ years n (%)**
High-need desert (IMD 1–3)	112	188,631	36,668 (19.4)	100,329 (53.2)	51,634 (27.4)
Other desert (IMD 4–10)	1,228	2,129,536	415,383 (19.5)	1,141,829 (53.6)	572,324 (26.9)
IMD, index of multiple deprivation.					

Summary counts (e.g., number of LSOAs and population affected) do not show whether high-need deserts are clustered or scattered, or where they occur in the country. Figure 1 shows that high-need dental deserts appear geographically clustered in non-metropolitan parts of England and are not concentrated in major inner-city conurbations. Using the Rural–Urban Classification 2021, high-need dental deserts were overwhelmingly rural: 97.0% (183,848/189,542, with only 3.0% (5,694/189,542)) in urban LSOAs. This indicates that dental deserts are not an inner-city phenomenon in this dataset. Cornwall, East Lindsey, King's Lynn and West Norfolk are the three local authorities with the greatest number of people in the high-need desert group (Table 3).

**Table 3:** The ten local authorities with the greatest number of people living in high-need dental deserts

**Rank**	**Local authority**	**High-need desert LSOAs n (%)**	**Population in high-need deserts (n)**	**% of high-need desert population**
1	Cornwall	19 (5.67)	33,926	17.9%
2	East Lindsey	15 (18.3)	25,519	13.5%
3	King's Lynn and West Norfolk	9 (9.78)	15,264	8.1%
4	Fenland	5 (8.93)	8,805	4.6%
5	East Riding of Yorkshire	6 (2.82)	8,575	4.5%
6	North Kesteven	3 (4.62)	5,878	3.1%
7	West Lindsey	4 (7.41)	5,549	2.9%
8	County Durham	4 (1.21)	5,439	2.9%
9	North Norfolk	3 (4.92)	4,547	2.4%
10	North Lincolnshire	3 (2.91)	4,533	2.4%
LSOA, lower layer super output area.				

In Table 3, high-need dental deserts were defined as LSOAs in IMD 1–3 with no NHS-listed provider within 5 km. Counts and populations refer to LSOAs meeting the high-need dental desert definition within each local authority. Percentages of the high-need desert population use the high-need desert population total of 189,542 as the denominator (see Table 1). The percentages shown in the ‘high-need desert LSOAs' column represent the percentage in each local authority that meet the high-need desert definition. Populations are from the IMD 2025 mid-2022 LSOA population denominator.

## Discussion

Most of the English population lived within short distances of an NHS-listed provider, while the 5 km threshold captured a smaller group experiencing more severe geographic isolation. Dental deserts were defined using straight-line distance from LSOA centroids to NHS-listed practices; this provides a consistent national screening metric but does not capture travel time, mode of travel, or local transport connectivity. Centroid-based distances may misrepresent access in geographically large, sparsely populated LSOAs because the geometric centroid can fall outside the LSOA boundary or in an uninhabited part of the area. Misclassification is therefore most likely for LSOAs close to the 5 km threshold and less likely for areas clearly above or below this cut-off. A sensitivity analysis recalculated distances using an alternative point guaranteed to lie within each LSOA boundary instead of the centroid. This changed the ‘no NHS dentist within 5 km' classification for 176 of 33,755 LSOAs (0.5%) overall. Within the high-need severe desert subgroup, seven of 112 LSOAs (6.3%) changed classification, affecting 13,352 of 189,542 people (7.0%). Overall, the main findings for high-need severe dental deserts were largely unchanged, indicating that conclusions were not strongly dependent on the choice of representative LSOA point.

This study measures severe proximity exclusion, i.e., LSOAs are flagged when there is no NHS-listed dental practice within 5 km, identifying places where geographic isolation is the dominant barrier. By contrast, other techniques such as floating catchment area methods estimate relative accessibility by balancing nearby provider supply against local population demand (often with distance-decay), thereby capturing competition and capacity constraints even where providers are geographically close. The Chief Medical Officer's report on the poor health outcomes of coastal communities noted that coastal deprivation can be masked when analysis is limited to larger administrative geographies.^4^ This supports the use of LSOA-level methods to identify small-area concentrations of combined need and access deficits.

The present analysis included only NHS dental provision, allowing recommendations on NHS commissioning, so private dental practices were not therefore included. The analysis only estimated the NHS access deserts, which may overstate scarcity where private provision is readily available. The findings estimate NHS-access deserts rather than total dental availability and may overstate geographic scarcity in areas where private provision fills any gaps. A useful next step would be to repeat the analysis using all Care Quality Commission-registered dental locations and compare NHS-only versus all-provider deserts.

Dental deserts (those without access to NHS dentists within 5 km) affects about 2.34 million people. In general, the deserts are more prevalent outside the most deprived IMD 1–3 communities because although deprivation can be concentrated in urban areas, NHS provision is generally higher there. It is the 189,542 people (0.33% of the English population) that lack NHS provision within 5km and are in the most deprived communities (IMD 1–3), that are the group best suited for targeted commissioning, outreach, or incentives. Although this represents a small proportion of England's population, it identifies communities facing combined social and geographic barriers, where targeted interventions are most likely to improve equity. The high-need severe desert subgroup was overwhelmingly rural and predominantly white (97.9%), consistent with the ethnic composition of rural LSOAs in IMD deciles 1–3 overall (97.2% white).‘Dental deserts' are also described in the literature in terms of constrained NHS capacity rather than complete absence of services.^5^ The present analysis used LSOAs with no NHS-listed dental provider within 5 km – capturing communities with marked geographic isolation from NHS dental provision and complementing capacity-based approaches.

Rahman *et al.*^6^ conducted a national spatial accessibility analysis of US dental clinics and found that rurality and socioeconomic deprivation were significant predictors of poor access to dental services. These underserved rural areas were predominantly inhabited by white populations. Similar challenges are evident in England, where recruiting and retaining dental professionals has become increasingly difficult. Evidence indicates that the situation is deteriorating, with several regions struggling to maintain a stable dental workforce.^7^ In Cornwall and Devon, for example, a survey of dental practices identified limited transport links, traffic congestion, high accommodation costs, and a lack of local training facilities as major disincentives to working in the area.^7^

The UK Government's 2025 consultation outcome proposes moderate reforms to the NHS dental contract intended to better align incentives with need.^8^ Measures include higher fees for unscheduled and urgent care, additional payments for patients with more complex treatment needs, and enhanced prevention payments for fissure sealants. While such changes may improve responsiveness and preventive delivery where services are already geographically accessible, this analysis indicates that severe access deficits are concentrated in non-metropolitan settings. Urban areas are generally well served by provider proximity, whereas deprived communities in geographically isolated rural LSOAs face a compounded access barrier that payment uplifts alone are unlikely to resolve, pointing to the need for more structural solutions in commissioning and service delivery. Edwards, Bogale and Gallagher's systematic review frames ‘dental deserts' as a health-systems challenge rather than a simple workforce shortfall.^9^ Drawing on 37 papers from 26 countries, it highlights a broad portfolio of access-improving interventions and emphasises that multi-component approaches are most likely to deliver sustained change. In this context, structural measures – such as targeted procurement of outreach and domiciliary services, support for satellite clinics, and workforce retention incentives – are more likely to succeed than single levers alone. This aligns with the present analysis, which identifies high-need severe dental deserts to help direct bundled strategies to communities where geographic isolation from NHS provision is most pronounced.

## Data Availability

Researchers wishing to have access to the data and R code should apply to the corresponding author (HD). This will be considered on a case-by-case basis, subject to institutional approval and data-sharing agreements.
